# Mr. Grove on Suspended Animation

**Published:** 1808-09

**Authors:** William Grove

**Affiliations:** St. John Street, Clerkenwell


### *To the Editors of the Medical and Physical Journal.*

GENTLEMEN,

AMONG the many accidental causes, tending to abridge the period of human existence, perhaps none are of more frequent occurrence than that arising from the sudden immersion in water, or drowning as it is termed, whereby life either becomes suspended or totally destroyed. When it is considered of how much national importance is the consideration of this subject to the inhabitants of a commercial state, whose welfare and security so much depend upon maritime superiority, it surely will not be thought presuming in me to offer a few observations upon the mode of treating persons in this situation. Thanks to the laudable zeal of a Society, so justly called the Humane, and to that individual, (Dr. Hawes) through whose philanthropic exertions so many valuable lives have been restored to their friends and to the community, little is left for me to remark upon the resusitative process; neither should I have troubled you with this article, had it not been with a view more strictly to enforce the doctrine inculcated by that excellent Institution.

In relating one or two practical facts, which occurred within the sphere of my own observation during the time I resided at Wapping; a situation which, from its local connection to the water, together with the extensive practice in which the gentleman with whom I then resided was engaged, offered frequent opportunities of attending to cases of this nature in the absence of the gentleman above alluded to. On Nov. 23, 1807, I had occasion to attend Thomas Harris, ætat. 40, of middle stature, and rather a plethoric habit; he was employed by the London Dock Company in the capacity of a watchman. In going his nocturnal rounds, being somewhat inebriated, he slipped his foot, and was precipitated into the water, where he remained upwards of twenty minutes ere he could be rescued from his perilous situation. When I saw him he presented to the view a lifeless body, having no signs of animation whatever; his physiognomy was bloated, and somewhat discoloured. I immediately proceeded to put in practice the means recommended by the Royal Humane Society, viz. Stripping the body and rubbing it dry with warm flannels, placing it in a room heated by a good fire, inflating the lungs, applying hot bricks to the soles of the feet, feet, &c. &c. These means were persisted in for upwards of an hour, without effect. It then occurred to me, that the warm bath, at a temperature of blood heat, would be the most likely mode of restoring him, as a more uniform and diffusible heat can be applied by this means than any other. The effect fully justified my expectation, for the man had not remained more than ten minutes in the bath before symptoms of returning life were manifested. By keeping up the degree of heat to the same temperature, and by means of fresh supplies of warm water, he was perfectly restored within half an hour. It is here necessary to premise, that when he was so far recovered as to be enabled to swallow, small doses of the volatile alkali were administered, which appeared materially to assist in his recovery. This medicine I have often employed, and with seemingly good effect.

Another Case, of a similar nature, occurred not long after in that of a Portuguese sailor, who fell overboard from a ship then lying in the river Thames, and remained under water nearly ten minutes. He was brought ashore, and I was desired to see him. The good effect which had resulted from the use of the warm bath in the former case determined me to have recourse to it more speedily in this. I therefore ordered one to be prepared as quickly as possible, and in the interval proceeded to put in practice the usual means of resuscitation, but without success. I then had him placed in the bath, at the same degree of temperature as in the former case, and, I am happy to say, with the same good effect.

From the season of the year in which these cases occurred, it should appear that cold contributed not a little to suspend the animal functions, aad that the bath was rendered more efficacious in consequence. However that may be, it is a remedy well deserving of attention; and where other means fail, ought, in my opinion, never to be omitted, if practicable.

I am, &c.

WILLIAM GROVE.

*St. John Street, Clerkenwell.*

